# Bitter Taste Disrupts Spatial Discrimination of Piperine-Evoked Burning Sensations: A Pilot Study

**DOI:** 10.3390/biology10090886

**Published:** 2021-09-08

**Authors:** David T. Liu, Gerold Besser, Karina Bayer, Bernhard Prem, Christian A. Mueller, Bertold Renner

**Affiliations:** 1 Department of Otorhinolaryngology, Head and Neck Surgery, Medical University of Vienna, 1090 Vienna, Austria; david.liu@meduniwien.ac.at (D.T.L.); gerold.besser@meduniwien.ac.at (G.B.); karina_1205@icloud.com (K.B.); bernhard.prem@meduniwien.ac.at (B.P.); 2 Institute of Experimental and Clinical Pharmacology and Toxicology, Friedrich-Alexander Universität Erlangen-Nürnberg, 91054 Erlangen, Germany; bertold.renner@tu-dresden.de; 3 Institute of Clinical Pharmacology, Medical Faculty Carl Gustav Carus, Technische Universität Dresden, 01307 Dresden, Germany

**Keywords:** chemesthesis, gustation, trigeminal, tongue, piperine, chemical senses

## Abstract

**Simple Summary:**

The chemical senses smell, taste, and trigeminal sense enable us to interact with the environment and play an essential role in protecting us from hazardous events. It is theorized that capsaicin and piperine not only elicit burning, but also bitter sensations through bitter taste-responding gustatory receptor cells that possess special channels. Similar psychophysiological responses to capsaicin and piperine suggest that bitter taste might also disrupt the spatial discrimination to piperine-induced burning sensations. Results showed that bitter taste disrupted the spatial discrimination of piperine-evoked burning sensations, providing further evidence for a qualitative similarity between burning and bitter sensations and the usefulness of chemical irritants in spatial discrimination tasks.

**Abstract:**

This study aimed to investigate the perceptual similarity between piperine-induced burning sensations and bitter taste using piperine-impregnated taste strips (PTS). This pilot study included 42 healthy participants. PTS of six ascending concentrations (1 mg, 5 mg, 10 mg, 15 mg, 20 mg, and 25 mg piperine/dL 96% ethanol) were presented at the anterior tongue, and participants rated perceived intensity and duration. Then, participants performed a spatial discrimination task in which they had to report which of the two strips presented to the anterior tongue contained an irritating stimulus when one strip was always a PTS while the other strip was impregnated with either a single taste quality (sweet or bitter) or a blank strip. Repeated measures one-way ANOVA revealed that burning sensations of higher concentrated PTS were perceived more intense and more prolonged compared to lower concentrated PTS. McNemar’s test showed that PTS were identified correctly significantly less often when presented with bitter strips compared to when presented with blank (*p* = 0.002) or sweet strips (*p* = 0.017). Our results showed that bitter taste disrupts the spatial discrimination of piperine-evoked burning sensations. PTS might serve as a basis for further studies on disease-specific patterns in chemosensory disorders.

## 1. Introduction

The chemical senses smell, taste, and trigeminal sense enable us to interact with the environment and play an essential role in protecting us from hazardous events [1,2,3]. While the senses of smell (olfactory system) and taste (gustatory system) enable us to perceive odors and gustatory stimuli (such as sweet, sour, salty, and bitter), the trigeminal sense (“trigeminal chemosense”) enables us to detect chemicals that activate receptors associated with pain, touch, and thermal perception [1,4,5,6]. Compared to the well-studied research area dedicated to identifying the mechanisms of chemesthesis (i.e., sensations that are perceived when trigeminal receptors are activated), relatively little is known about the interaction between trigeminal and gustatory sensations. Besides pioneering studies in the 1980s [7,8,9,10], it was only during the past 20 years that researchers started to investigate irritant-taste interactions [11,12,13]. Nevertheless, these pioneering studies on the oral perception of chemical irritants—such as capsaicin and piperine—provided the first evidence that these irritants elicit not only chemesthetic sensations of burning and stinging but also gustatory sensations such as salty and bitter [8,10]. The findings that capsaicin elicits not only burning sensations but also a bitter taste have been confirmed more recently, providing further evidence for a qualitative similarity [11,12,13]. Lim and Green [14] then investigated the effect of capsaicin on the tongue tip’s spatial discrimination of taste stimuli—i.e., identifying the taste stimuli (sweet, sour, salty, or bitter) when presented with two blank swabs containing water and identifying the taste stimuli when capsaicin was additionally present on all three swabs—and showed that burning sensations of capsaicin only disrupted the spatial discrimination for bitter, but not for sweet, sour, or salty taste.

Considering the role of capsaicin as a prototypical chemesthetic stimulus [5], it is thus not surprising that this irritant has been used for most studies to investigate further the qualitative similarity between trigeminal and gustatory (especially bitter) sensations [11,12,13,15]. Although previous studies already assessed psychophysiological responses to piperine, the pungent compound of black pepper within the oropharyngeal region [7,9,13,15,16,17], its role and effect on spatial discrimination of taste stimuli still need further investigation. Such comparison might provide further evidence for the qualitative similarity between burning and bitter sensations and the usefulness of chemical irritants in spatial discrimination tasks.

Transient receptor potential (TRP) channels are biological sensors that contribute to our oral somatosensory perception [18]. TRP channels are divided into subfamilies and can be differentiated based on their molecular structure [19,20]. Capsaicin, which is best known for causing burning sensations, is an agonist of the TRP channel subfamily vanilloid member 1 (TRPV1) [21]. Similarly, piperine, the pungent component of black pepper, serves as an agonist for TRPV1 and TRP channel subfamily ankyrin 1 (A1) receptors [22]. While capsaicin has already found its way into the clinical routine as a valuable pharmacological agent and oral trigeminal threshold test, the first clinical trials on the analgesic effect of TRPA1 receptors-antagonists for neuropathic pain have only recently started [19,23]. Indeed, it has been shown that patients with burning mouth syndrome (BMS)—a condition characterized as pain and burning sensations of the oral cavity—exhibit higher oral trigeminal thresholds measured using an above-mentioned capsaicin-based taste strips test [24].

Previous studies on the oral perception of chemical irritants provided a clearer picture about the dose-, regional-, and stimuli-dependent effect of perceived irritation after trigeminal stimulation based on capsaicin and piperine [8,10,16,17,25,26,27,28,29,30,31,32,33,34]. Similarly, the effect of capsaicin to elicit bitter taste sensations has also been reported previously [11,12,13,14,15,35]. Green and Schullery [11] found that approximately half of the investigated subjects reported bitter taste sensations following capsaicin stimulation to the circumvallate region. This finding was later confirmed and expanded for piperine and zingerone, irritants related to capsaicin [13]. The authors hypothesized that capsaicin, piperine, and zingerone might elicit bitter sensations through bitter taste-responding gustatory-receptor cells that possess the TRPV1s channel. The possibility that capsaicin, piperine, and zingerone directly stimulate bitter taste receptors (T2R) has also been proposed as an alternative explanation for this qualitative closeness. Similar psychophysiological responses to capsaicin and piperine suggest that bitter taste might also disrupt the spatial discrimination to piperine-induced burning sensations.

Thus, this study aimed (i) to investigate whether bitter taste also increases the difficulty for spatial discrimination of piperine-evoked burning sensations (as shown for capsaicin), (ii) to assess the dose-dependent effect of piperine-impregnated taste strips on psychophysical responses (duration, intensity, and beginning of chemical irritation), and (iii) to evaluate the usefulness of PTS as a trigeminal test method to assess spatial discrimination ability.

## 2. Materials and Methods

### 2.1. Subjects

This study followed a longitudinal, mono-centered, and experimental design. Healthy subjects were prospectively recruited through advertisements attached to messaging boards at the General Hospital of Vienna/Medical University of Vienna between March 2018 and December 2019. All participants were 18 or older and agreed to refrain from ingesting anything except standard tap water one hour before participation. We included healthy participants without disorders related to the chemosensory functions smell and taste (i.e., anosmia and severe dysgeusia). Participants were asked for current or past conditions related to olfactory or gustatory dysfunction (head trauma, acute oral or dental infections, tumors or surgeries of the head and neck, or regular intake of medication affecting the sense of taste) and self-rated the chemosensory functions smell, taste, and flavor perception. Furthermore, all participants underwent psychophysical olfactory and gustatory testing, as previous studies showed that there might exist differences between self-reported and measured chemosensory functions [36,37,38]. We excluded all participants who regularly consume black pepper or spicy food (“I consume spicy food (i.e., chili and black pepper) more than three times per week”).

### 2.2. Psychophysical Olfactory and Gustatory Testing

Before experimental sessions, all participants were asked to rate their self-perceived sense of smell, taste, and flavor perception on a numeric rating scale ranging from 1 to 10 (left-hand end, 1 = no perception; right-hand end, 10 = perfect perception) and underwent olfactory and gustatory testing utilizing validated psychophysical test methods. Olfactory testing was performed using the 16-item Sniffin’ Sticks Identification test (Burghart Messtechnik, Holm, Germany), while gustatory testing was performed using the Taste Strips Test (TST, Burghart Messtechnik, Holm, Germany) [39,40,41]. The TST utilizes taste strips soaked with four different concentrations of sweet (sucrose), sour (citric acid), salty (sodium chloride), and bitter (quinine hydrochloride) solutions. The exact testing procedure is described elsewhere [41].

### 2.3. Stimuli

Piperine impregnated taste strips (further termed “PTS”) were prepared according to the original taste strips protocol [41]. Piperine (Sigma Aldrich, #P49007) was diluted in ethanol (96%) and six different piperine concentrations were prepared: 1 mg (PTS1), 5 mg (PTS5), 10 mg (PTS10), 15 mg (PTS15), 20 mg (PTS20) and 25 mg (PTS25) piperine/dL 96% ethanol. Paper strips with a length of 8 cm and 2 cm^2^ area to be impregnated were then soaked in these solutions and dried on a slowly rotating wheel. Paper strips without impregnation were used as blank strips.

### 2.4. Procedures

#### 2.4.1. Experiment 1: Psychophysiological Responses to PTS

The first experiment of this study was designed to (i) study the concentration-dependent effect of PTS on psychophysiological responses (beginning, duration, and intensity of irritation) and (ii) determine the PTS concentration that results in burning and stinging sensations in at least 90% of participants. Participants were instructed to rinse their mouth with room temperature tap water before each test round. The first experiment consisted of one session with eight trials. Each trial consisted of placing one PTS or one blank taste strip at the tip of the tongue for 15 s (Table 1). Subjects were told that the presented strip was either blank or impregnated with piperine. They were further instructed to raise their hand when an irritation was noticed and to indicate for how long they perceived this irritation by laying down the hand. Both the start and the end of irritation were noted using a timer. Furthermore, participants were instructed to keep their mouths slightly open during the testing procedure so that the taste strip did not touch the hard palate. After laying down the hand, subjects were asked to rate the maximum perceived intensity on a visual analogue scale ranging from 1 to 10 (left-hand end, 1 = minimal irritation; right-hand end, 10 = maximal irritation) and to characterize the irritation as “stinging” or “burning”. The interstimulus interval (ISI) was started after participants laid down their hands and were set at 30 s for lower concentrations (1, 5, 10, and 15 mg/dL) and at 60 s for higher concentrations (20 and 25 mg/dL). In case of no perceived irritation, ISI was started after the strip was removed. An experimenter was assigned to each participant and recorded all responses.

#### 2.4.2. Experiment 2: Spatial Discrimination between Gustatory and Piperine-Evoked Burning Sensations

Previous studies on trigeminal stimuli revealed that capsaicin, piperine, and zingerone might induce bitter sensations when presented orally [11]. Moreover, it was also shown that capsaicin only increases the difficulty of spatial discrimination for bitter (quinine sulfate), but not for sweet (sucrose), salty (NaCl), or sour (citric acid) taste [14]. Therefore, the second experiment was designed to study whether bitter taste also increases the difficulty of spatial discrimination for piperine-evoked burning sensations. Bitter taste strips impregnated with the highest concentration of a quinine hydrochloride solution [41] were chosen as bitter stimuli. Sweet strips impregnated with the highest concentration of a sucrose solution [41] and taste strips without impregnation were chosen as a placebo control. Subjects were invited to two test sessions with at least 24 h in between sessions. In comparison to previous studies, we used a test protocol similar to the lateralization task to identify pure odorants [42]. Therefore, we presented the target irritation on one side and the blank/taste stimulus on the other side of the tongue. Each session included nine trials in which two strips (one PTS and either one sweet, bitter, or blank strip) were placed simultaneously at the left and right anterior tongue for 15 s. The test protocol is summarized in Table 2. After removing both strips, the subjects’ task was to correctly identify the tongue’s side on which a chemical irritation was perceivable (two-alternative, forced-choice procedure). Participants were instructed to keep their mouths slightly open during the testing procedure (to avoid strips touching the hard palate) and rinse their mouth with room temperature tap water before the strip presentation. The number of correctly identified PTS was counted for each sweet, bitter, or placebo stimuli separately, resulting in a score ranging from 0 to 3. The ISI was set at 60 s, and a timer was started after participants indicated that no irritation was perceivable. PTS of 15 mg/dL were selected based on results from Experiment 1 (representing a suprathreshold PTS concentration). To verify results from the first session, a subset of participants repeated Experiment 2 in a second session.

### 2.5. Statistical Analysis

Continuous data are presented as mean (standard deviation, SD) or median (IQR: lower-upper bound). Categorical data are presented as number (percentage,%). The normality of data was visualized based on histograms and Q-Q plots. Repeated measures one-way analysis of variance (rm-ANOVA) with Tukey’s post-hoc test was performed to depict the effect of PTS concentration (1, 5, 10, 15, 20, and 25) on perceived (i) duration (in seconds) and (ii) intensity (numeric number scale) in Experiment 1. To depict whether PTS are identified less often as a chemical irritant when presented together with a bitter taste (compared to sweet and blank taste strips) in the first session of Experiment 2, we summed participants’ number of correctly identified PTS for each of the three strip types, resulting in a score ranging from 0 to 90 (30 participants, three trials for each of the three strip types; see Table 2). The number of correctly identified PTS were then compared between (i) the bitter and the sweet condition and (ii) the bitter and the blank condition using McNemar’s test. Finally, to assess the reliability and agreement of the spatial discrimination task (Experiment 2, session one), we employed the Bland–Altman statistical method and included results of both sessions from those participants who were revisited in Experiment 2. Based on the number of correctly identified PTS between both sessions, we calculated the bias representing the mean difference of correctly identified PTS between both sessions for each stimulus. Alpha level was set at 0.05 for the rm-ANOVA analyses and at 0.025 for McNemar’s test to account for multiple comparisons. Statistical analysis and graphical visualization were performed using SPSS (SPSS version 26.0 for Windows; IBM Corp., Armonk, NY, USA) and GraphPad Prism 8.4.2 (GraphPad Software, Inc., La Jolla, CA, USA).

## 3. Results

### 3.1. Participants

The study included a total of 42 healthy subjects (29 women, mean age/SD = 33.5/15.7 years) in two independent experiments: Experiment 1, with 12 participants, six women, mean age/SD = 35.1/16.5 years and Experiment 2, with 30 participants, 23 women, mean age/SD: 37.4/18.1 years. Reliability of Experiment 2 was assessed in 15 participants who revisited with a mean duration/SD of 8/7 days in-between sessions: 11 women, mean age/SD = 22.0/0.6 years.

### 3.2. Olfactory and Gustatory Test Results Correspond to the Self-Perceived Functions

Olfactory test scores revealed that all participants scored within the normosmic or mild hyposmic range (Sniffin’ Sticks identification test score, mean/SD = 12.8/1.9). Similarly, all participants scored within the normogeusic or mild dysgeusic range (taste strips test score, mean/SD = 12.0/2.4). Likewise, self-ratings of chemosensory function smell, taste, and flavor perception were similar to objective test scores: for the self-perceived sense of smell, mean/SD = 7.2/1.9; for taste, mean/SD = 7.4/1.6; and for flavor, mean/SD = 6.1/2.0.

### 3.3. Increasing PTS Concentrations Lead to Higher Perceived Intensity and Longer Duration of Irritation

Descriptive analysis revealed that higher PTS concentrations more frequently resulted in burning and stinging perceptions (Table 3). PTS15 was perceived as chemically irritating by all participants.

**Figure 1:**
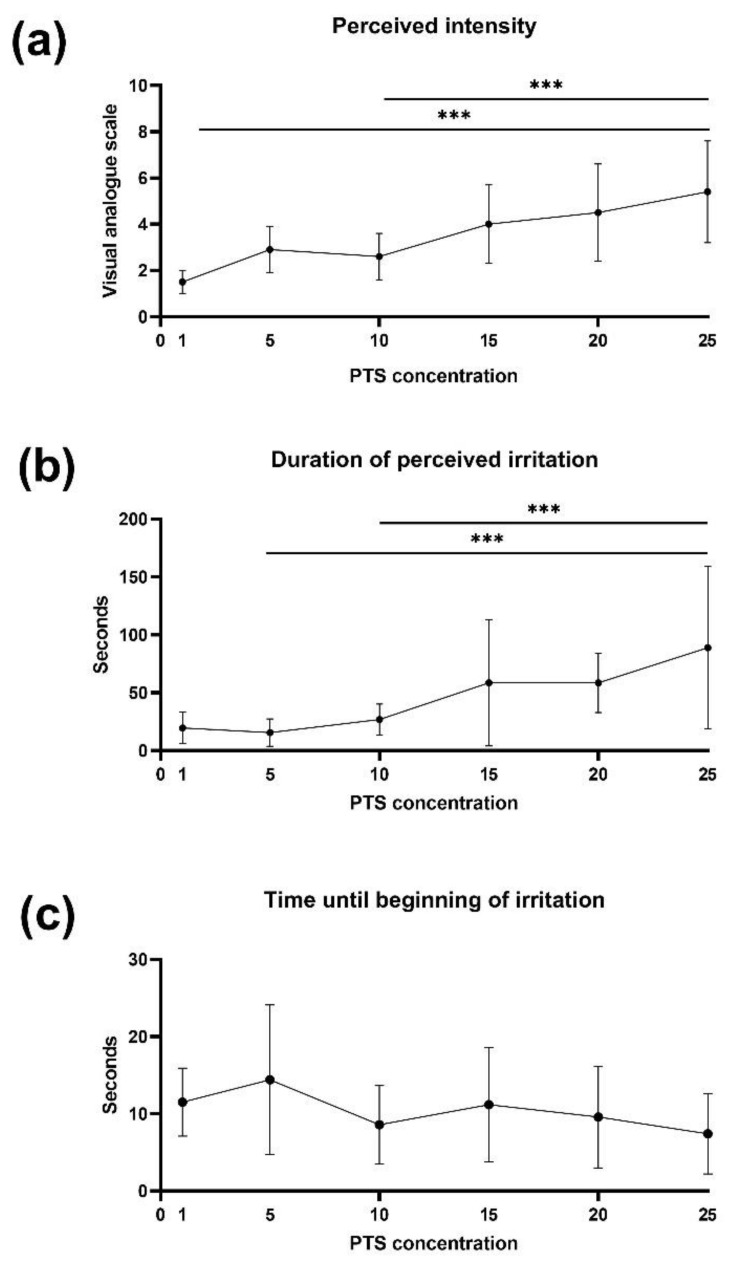
Psychophysiological responses to piperine impregnated taste strips (PTS) of Experiment 1 (n = 12). (**a**) The intensity of perceived irritation. (**b**) The duration (seconds) of perceived irritation. (**c**) The duration (seconds) until an irritation was perceived. The middle point represents the mean, and the error bars mark the standard deviation. Groups were compared using the rm-ANOVA test with post-hoc Tukey’s test. *** *p* < 0.05.

Repeated measures one-way ANOVA revealed a main effect of PTS concentration on perceived intensity [F(3.474, 38.21) = 13.26, *p* < 0.0001] and duration of irritation [F(1.740, 19.14) = 9.234, *p* = 0.002]. On the contrary, one-way rm-ANOVA revealed no significant differences in the beginning of irritation between different concentrations of PTS [F(5.66) = 1.278, *p* = 0.28]. Tukey’s post-hoc test showed that higher concentrated PTS were perceived as more intense and longer than lower concentrated PTS (Figure 1).

### 3.4. Bitter Taste Disrupts Spatial Discrimination of Piperine-Evoked Burning Sensations

To test the hypothesis of whether bitter taste disrupts spatial discrimination of piperine-evoked burning sensations—i.e., piperine-evoked burning sensations are identified less often when presented simultaneously with bitter strips compared to sweet and blank placebo strips—we compared the number of correctly identified PTS when presented with a bitter taste to (i) the sweet and (ii) the placebo condition using McNemar’s test.

When PTS were presented with blank placebo strips, burning sensations were correctly identified in 79/90 (87.8%) trials. Similarly, PTS were correctly identified in 76/90 (84.4%) trials when presented with sweet taste strips. On the contrary, PTS were only correctly identified in 61/90 (67.8%) of trials when presented with bitter strips. McNemar’s test revealed that PTS were identified significantly less often correctly when presented with bitter strips compared to when presented with (i) blank (exact *p* = 0.002) or (ii) sweet strips (exact *p* = 0.017).

### 3.5. Spatial-Discrimination between Piperine-Evoked Burning Sensations and Gustatory Stimuli Is Reliable

**Figure 2:**
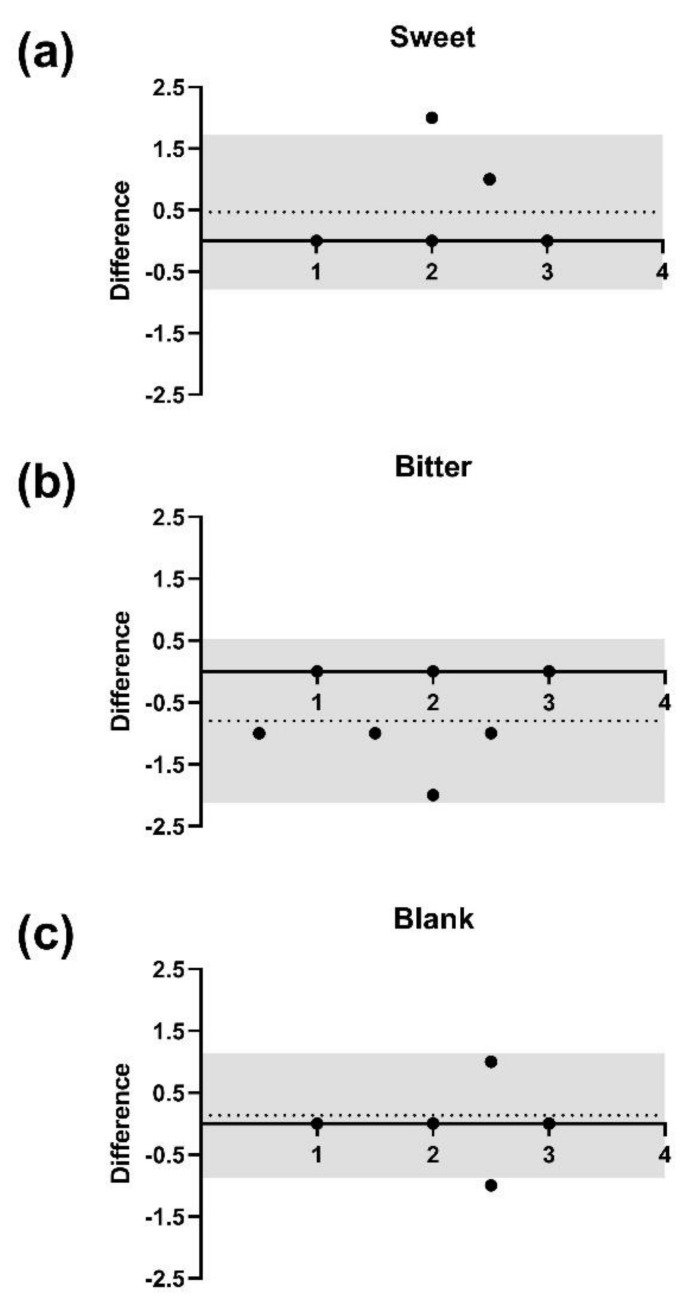
Bland–Altman plots for the reliability for the spatial discrimination task between two experimental sessions (n = 15). The vertical axis shows the differences in correctly identified piperine taste strips (PTS) between the first and second sessions. The horizontal axis shows the average number of correctly identified PTS between both sessions for each stimulus separately. The 95% limits of agreement are indicated within the grey area, the horizontal dotted line indicates the bias (mean difference). (**a**) Sweet taste strips compared to PTS. (**b**) Bitter taste strips compared to PTS. (**c**) Blank taste strips compared to PTS.

We were next interested in determining whether results from the spatial-discrimination task of Experiment 2 were reliable. Therefore, we invited a subset of 15 participants from the first session with at least 24 h in between sessions. The intra-individual reliability of the discrimination task was then evaluated for each stimulus separately based on the Bland–Altman statistical method (Figure 2).

The bias of nearly zero for each stimulus indicated neither a proportional nor a systemic error: for sweet, bias (0.47) and 95% limits of agreements (LOA) from −0.79 to 1.72 (Figure 2a); for bitter, bias (−0.80) and 95% LOA from −2.13 to 0.53 (Figure 2b); and for blank, bias (0.13) and 95% LOA from −0.88 to 1.15 (Figure 2c).

## 4. Discussion

Although previous studies provided evidence that capsaicin-evoked burning sensations and bitter taste are perceptually similar and confusable, there remains a gap of knowledge about whether this perceptual similarity also applies to piperine-evoked burning sensations and bitter taste. The present study demonstrates that piperine-induced burning sensations and bitter taste perceptions are perceptually similar. Moreover, we confirm previously published literature demonstrating the concentration-dependent effect of orally presented piperine on psychophysiological responses [16]. Finally, we provide evidence for the reliability of results from the spatial discrimination task between piperine-evoked burning sensations and gustatory stimuli based on PTS.

Considering the pilot study design of the current investigation, we decided to measure smell and taste based on validated psychophysical methods before experimental sessions to ensure that our cohort was homogenous in olfactory and gustatory functions. Previous studies have shown that there might exist striking differences between self-perceived and measured olfactory and gustatory functions as mentioned in the methods [36,37,38,43,44]. In those studies, the authors reported that almost one-third of healthy subjects without self-reported olfactory dysfunction score within the hyposmic or even anosmic range. Similarly, a study on self-reported gustatory function in patients after tonsillectomy also revealed that there might be differences between self-reported and measured gustatory function [43]. Furthermore, it has also been shown that patients with smell loss demonstrate decreased trigeminal sensitivity [45]. Noteworthy, although there might exist differences between measured and self-reported gustatory function in patients with chemosensory disorders, one previous study concluded that the majority of patients who explicitly report no problems related to the sense of taste usually do not exhibit gustatory dysfunction [44]. The finding that olfactory and gustatory test results corresponded with self-perceived chemosensory functions in our cohort of healthy participants was, therefore, not unexpected. Nonetheless, screening of olfactory and gustatory function—even in healthy participants before participation—might further minimize the potential bias concerning differences between self-reported and measured senses of smell and taste.

Our findings of the main effect of piperine concentration on perceived intensity have also been reported previously [16]. The authors found a main effect of concentration and duration of perceived irritation on perceived intensity. Compared with capsaicin, the author found no main effect of the stimulus location (i.e., anterior or posterior tongue) for piperine on perceived intensity. The authors hypothesized that capsaicin elicits larger spatial differences in responses within the oropharyngeal region compared to piperine. Based on these findings, we also hypothesized that higher concentrated PTS result in higher perceived intensity of irritation—which we found to be the case. We also found no significant differences between different PTS concentrations regarding the time until the beginning of perceived irritation. This finding was not unexpected, since a previous study on psychophysical responses to orally presented piperine also reported no differences in time until the beginning of irritation between different piperine concentrations [10].

It is not yet possible to fully explain the qualitative similarity between burning and bitter sensations. Regarding central gustatory pathways involved in multisensory processing of gustatory and somatosensory information, previous work suggested that the gustatory cortex plays a major role in the multisensory integration of olfactory, gustatory, and somatosensory information during eating and drinking [46,47,48,49,50]. Interestingly, there is an ongoing debate about whether single taste qualities are spatially organized within the mammalian primary taste cortex. While some studies provided evidence of a taste-specific activation pattern of neurons [51,52], other studies have shown otherwise [53,54,55,56,57]. One explanation for the present findings—that only bitter taste disrupted spatial discrimination of piperine-evoked burning sensations—relates to the ability of chemesthetic stimuli to induce bitter taste in some individuals, which might have increased the difficulty of the spatial discrimination task in terms of perceptual similarity [15,47,48,49,50]. Based on this hypothesis, one might reasonably suggest that the qualitative similarity between bitter and burning sensations is most pronounced when using weakly concentrated bitter solutions, since studies have shown that capsaicin-evoked burning sensations were mainly perceived as weakly bitter. However, a more recent psychophysiological study on the qualitative similarity between bitter taste induced by quinine sulfate and burning sensations induced by capsaicin failed to find evidence for this hypothesis, as capsaicin was only able to disrupt the spatial discrimination at the highest presented concentration of quinine sulfate [14]. It is unknown how the effect of bitter taste on the spatial discrimination of chemesthethic stimuli (i.e., piperine or capsaicin) may potentially differ depending on the presented concentrations of both stimuli. This should be revisited in future work to advance our understanding of similarities and differences in the dose-dependent effect of piperine and capsaicin in perceived bitterness and oral irritation.

We also found that the spatial discrimination task results were reliable, showing only a small intra-individual variability over time. Interestingly, the smallest bias between both sessions—representing the mean difference of correctly identified PTS between the first and second session—was found for the discrimination task with placebo blank strips. This result indicates that a true gustatory stimulus might increase the spatial discrimination task’s difficulty, resulting in higher intra-individual variability. The test-retest reliability is also essential in terms of the clinical relevance of the PTS discrimination task. Further investigations based on the proposed method may be implemented quickly, as the manufacturing process of PTS is relatively simple. The finding that PTS15 represented a threshold concentration that elicited burning sensations in most participants provides a basis for future studies that focus on the oral trigeminal sensitivity in health and disease. The role of trigeminal tests and oral sensitivity in patients with BMS has been outlined previously [24,58,59]. The authors provided the first evidence that the oral pain threshold is higher in patients with BMS than healthy controls [58]. The authors proposed that the degeneration of peripheral nerves may induce changes in pain perception’s central processing. Similarly, Just et al. also showed that lateralized oral trigeminal perception is decreased in patients with BMS compared to healthy subjects [24]. However, they also noticed that further research is needed to elucidate BMS patients’ differences compared to healthy controls concerning threshold and suprathreshold chemesthetic stimuli.

The present study uses a mono-centered and experimental design to investigate the effect of PTS on psychophysiological responses. However, this study also has limitations. First, we mainly included young female subjects, while a study including male subjects older in age may have elucidated age- and gender-related differences in the ability to discriminate between burning and gustatory sensations spatially. Second, the sample sizes of our experiments were small. Therefore, results might not be representative of the entire population considering the inter-individual differences. Third, the evaluation of only six different piperine concentrations might have overestimated the true detection threshold for PTS. Further studies are needed to elucidate whether PTS15 represents the true detection threshold of the irritation or only the bitterness of piperine. Fourth, our study does not provide evidence for differences in piperine sensations between different parts of the tongue, nor is our study intended to. Nonetheless, our results should also be validated at the posterior regions of the tongue. Lastly, this was a pilot study only including healthy subjects, while a study including patients with BMS may have further revealed disease-specific patterns. Our results are nevertheless essential to serve as motivation for pursuing such studies in the future.

## 5. Conclusions

In summary, this study expands our current knowledge of perceptual responses to piperine in three important ways. First, piperine-evoked burning sensations were identified less often when presented with a bitter taste (compared to sweet and placebo) in our cohort of healthy participants. Second, it confirms previously published literature showing that psychophysiological responses to orally presented piperine follow a concentration-dependent relationship. Third, it provides evidence that results from the spatial discrimination task between burning and gustatory sensations using PTS are reliable. Therefore, the proposed discrimination task might serve as a useful trigeminal test method to assess spatial discrimination ability and further reveal disease-specific patterns in chemosensory disorders such as BMS.

## Figures and Tables

**Table 1:** The order in which the piperine impregnated taste strips and blanks were presented.

Order	Strips	Concentration
1	Piperine	1 mg/dL
2	Piperine	5 mg/dL
3	Piperine	10 mg/dL
4	Piperine	15 mg/dL
5	Blank	-
6	Piperine	20 mg/dL
7	Blank	-
8	Piperine	25 mg/dL

**Table 2:** The test protocol used for the strips’ presentation in Experiment 2.

Trial	Left Anterior Tongue	Right Anterior Tongue
1	Sweet	Piperine
2	Bitter	Piperine
3	Piperine	Blank
4	Sweet	Piperine
5	Piperine	Bitter
6	Piperine	Blank
7	Sweet	Piperine
8	Piperine	Bitter
9	Blank	Piperine

**Table 3:** Psychophysiological responses to piperine impregnated taste strips (PTS) and blanks of Experiment 1 (n = 12).

PTS	Irritation Perceived, N	Delay in Seconds, Mean/SD	Duration in Seconds, Mean/SD	Intensity in Estimation Units, Mean/SD	Burning, N	Stinging, N
PTS1	4	4.2/6.4	6.5/12.6	0.5/0.8	1	3
PTS5	7	14.4/10.1	9.1/12.4	1.7/1.7	4	3
PTS10	10	8.6/5.4	22.4/16.4	2.2/1.4	6	4
PTS15	12	11.2/7.7	58.5/56.9	3.8/2.1	8	4
Blank 1	6	5.3/7.4	13.7/21.3	1.3/1.8	4	2
PTS20	11	9.4/6.9	52.8/30.6	4.1/2.4	9	2
Blank 2	6	10.0/10.2	12.9/19.8	1.2/1.3	3	3
PTS25	11	7.4/5.4	80.1/74.6	4.9/2.7	9	2

## Data Availability

The data presented in this study are available on request from the corresponding author. The data are not publicly available due to guidelines of the Institutional Review Board.
